# Supplementary material for: Achieving universal sanitation in Ghana: An analysis of key drivers of toilet ownership among property owners in Urban areas
Source: PLoS One. 2025 Jan 16;20(1):e0307729. doi: 10.1371/journal.pone.0307729 (PMC11737778; doi:10.1371/journal.pone.0307729)
Supplement: S1 Table — (DOCX) [file pone.0307729.s001.docx]

**S1 Table: Types of toilet facilities owned by property owners**

| **Type** | **Akuapem North**  **(%)** | **Ga West**  **(%)** | **Kumasi**  **(%)** |
| --- | --- | --- | --- |
| WC with septic tank | 43.3 | 53.8 | 75.6 |
| K(VIP) toilet | 42.7 | 20.5 | 11.4 |
| Traditional pit latrine | 8.1 | 11.9 | 1.9 |
| Pour flush toilet | 3.6 | 6.7 | 3.6 |
| Biodigester toilet | 0.7 | 3.5 | -- |
| Aqua privy | 0.3 | 3.5 | -- |
| Bucket latrine | 1.3 | -- | -- |
| Sewer | -- | -- | 7.5 |
| **Total** | **100.0** | **100.0** | **100.0** |
