# Supplementary material for: Achieving universal sanitation in Ghana: An analysis of key drivers of toilet ownership among property owners in Urban areas
Source: PLoS One. 2025 Jan 16;20(1):e0307729. doi: 10.1371/journal.pone.0307729 (PMC11737778; doi:10.1371/journal.pone.0307729)
Supplement: S2 Table — (DOCX) [file pone.0307729.s002.docx]

**S2 Table: Where households without toilet facilities defecate**

| **Where** | **Akuapem North**  **(%)** | **Ga West**  **(%)** | **Kumasi**  **(%)** |
| --- | --- | --- | --- |
| Public toilet | 69.6 | 72.1 | 97.4 |
| Use neighbour’s toilet | 18.6 | 17.1 | 1.7 |
| Open defecation | 8.8 | 9.9 | -- |
| Both open defecation and public toilet | 2.9 | 0.9 | 0.9 |
| **Total** | **100.0** | **100.0** | **100.0** |
