# Supplementary material for: Achieving universal sanitation in Ghana: An analysis of key drivers of toilet ownership among property owners in Urban areas
Source: PLoS One. 2025 Jan 16;20(1):e0307729. doi: 10.1371/journal.pone.0307729 (PMC11737778; doi:10.1371/journal.pone.0307729)
Supplement: S3 Table — (DOCX) [file pone.0307729.s003.docx]

**S3 Table: Respondent's Level of Satisfaction with Public Toilets**

| **Level of Satisfaction** | **Akuapem North**  **(%)** | **Ga West**  **(%)** | **Kumasi**  **(%)** |
| --- | --- | --- | --- |
| Very Satisfied | 7.1 | 5.0 | 6.9 |
| Satisfied | 22.9 | 40.0 | 65.5 |
| Neutral | 14.3 | 30.0 | 8.6 |
| Dissatisfied | 35.7 | 25.0 | 15.5 |
| Very Dissatisfied | 20.0 | 0.0 | 3.4 |
| **Total** | **100.0** | **100.0** | **100.0** |
