# Supplementary material for: Achieving universal sanitation in Ghana: An analysis of key drivers of toilet ownership among property owners in Urban areas
Source: PLoS One. 2025 Jan 16;20(1):e0307729. doi: 10.1371/journal.pone.0307729 (PMC11737778; doi:10.1371/journal.pone.0307729)
Supplement: Attachment — Submitted filename: Achieving Universal Sanitation in Ghana_Reviewers Comments.docx [file pone.0307729.s004.docx]

**Achieving Universal Sanitation in Ghana: An Analysis of Key Drivers of Toilet Ownership Among Property Owners in Urban Areas.**

**Comments**

The paper addresses an important public health issue in urban Ghana, focusing on the determinants of household toilet ownership.

The manuscript is generally well-structured, with a clear flow from the introduction through to the conclusion. However, in my review I did not see the introduction section. A technical error, I guess. The introduction could benefit from a more comprehensive literature review that situates the study within the context of existing research on urban sanitation in Ghana and other similar contexts – If the authors have not adequately done so.

The authors should provide more justification for the choice of the three urban districts selected for the study. Were these districts chosen because they are representative of urban areas in Ghana, or were there other reasons?

It would also be beneficial to discuss any potential limitations related to the data collection process, such as response bias or the accuracy of self-reported data on household income and building characteristics.

The discussion appropriately connects the results to the broader context of urban sanitation in Ghana. However, it would benefit from a more critical analysis of the findings. For instance, the paper suggests that "enforcing building regulations requiring toilet facilities in all properties is crucial." Could the authors provide more concrete examples of how such regulations could be enforced in practice?

The suggestion for "innovative sanitation financing interventions" is a good one, but the paper could delve deeper into what these interventions might look like, particularly in the context of urban Ghana where financial constraints are a significant barrier.

The conclusion succinctly summarizes the key findings and recommendations. However, the authors could enhance the impact of their conclusions by explicitly linking them to the Sustainable Development Goals (SDGs), particularly SDG 6 (Clean Water and Sanitation).

Authors indicated that “The survey was supplemented with field notebooks and cameras to aid direct field observations.” I did not see results reflective of that, or at least images/ pictures of observations from the field.

In the discussion from line 6756 provide more specific details of the studies you are engaging with……eg. For example, a study in Ghana found that…. where exactly in Ghana?…

**Overall Recommendation:** The paper addresses a critical issue in urban sanitation and provides valuable insights into the determinants of toilet ownership among property owners in Ghana. With some revisions of suggested areas for improvement, the manuscript could make a significant contribution to the field and be a valuable resource for policymakers. I recommend **minor revisions** before the manuscript is considered for publication.
