# Supplementary material for: Achieving universal sanitation in Ghana: An analysis of key drivers of toilet ownership among property owners in Urban areas
Source: PLoS One. 2025 Jan 16;20(1):e0307729. doi: 10.1371/journal.pone.0307729 (PMC11737778; doi:10.1371/journal.pone.0307729)
Supplement: Attachment — Submitted filename: Response to Reviewers.docx [file pone.0307729.s005.docx]

**Authors Response to Reviewer’s Comment**

**Achieving Universal Sanitation in Ghana: An Analysis of Key Drivers of Toilet Ownership Among Property Owners in Urban Areas.**

**Comments**

The paper addresses an important public health issue in urban Ghana, focusing on the determinants of household toilet ownership.

1. The manuscript is generally well-structured, with a clear flow from the introduction through to the conclusion. However, in my review, I did not see the introduction section. A technical error, I guess. The introduction could benefit from a more comprehensive literature review that situates the study within the context of existing research on urban sanitation in Ghana and other similar contexts – If the authors have not adequately done so.

***Response:*** *We sincerely appreciate your thoughtful feedback and apologize for the technical error. The introduction was indeed included in the original submission, and we have ensured that it is now properly visible in the revised manuscript. We believe we have undertaken a comprehensive literature review to situate the study within the broader context of urban sanitation in Ghana and similar contexts. We have thoroughly gone through the introduction to improve the flow and coherence of our argument as per your suggestion.*

2. The authors should provide more justification for the choice of the three urban districts selected for the study. Were these districts chosen because they are representative of urban areas in Ghana, or were there other reasons?

***Response:*** *Thank you for highlighting this. We selected the three districts (Kumasi Metropolitan Assembly, Ga West Municipal, and Akuapem North Municipal) using a random sampling method, and we have now included a detailed explanation of this process in the methodology section. We have also provided justification in the introduction section that these districts, though randomly chosen, represent different levels of urbanization in Ghana, thus providing a diverse urban context for the study.*

3. It would also be beneficial to discuss any potential limitations related to the data collection process, such as response bias or the accuracy of self-reported data on household income and building characteristics.

***Response:*** *We acknowledge the importance of discussing limitations and have now expanded the section on data limitations. Specifically, we have addressed potential response biases and the accuracy of self-reported data.*

4. The discussion appropriately connects the results to the broader context of urban sanitation in Ghana. However, it would benefit from a more critical analysis of the findings. For instance, the paper suggests that "enforcing building regulations requiring toilet facilities in all properties is crucial." Could the authors provide more concrete examples of how such regulations could be enforced in practice?

***Response:*** *We appreciate this insightful comment. We have expanded our discussion to provide more concrete examples of how building regulations requiring toilet facilities in all properties could be enforced in practice. For instance, we now discuss specific mechanisms such as routine building inspections, stricter penalties for non-compliance, and the potential for leveraging local government partnerships to ensure adherence.*

5. The suggestion for "innovative sanitation financing interventions" is a good one, but the paper could delve deeper into what these interventions might look like, particularly in the context of urban Ghana where financial constraints are a significant barrier.

*Response: Thank you for this suggestion. We have expanded our discussion on potential innovative sanitation financing interventions, particularly within the context of urban Ghana. We now provide examples such as microfinance schemes for toilet construction and revolving fund to overcome financial barriers.*

6. The conclusion succinctly summarizes the key findings and recommendations. However, the authors could enhance the impact of their conclusions by explicitly linking them to the Sustainable Development Goals (SDGs), particularly SDG 6 (Clean Water and Sanitation).

***Response:*** *We agree with this recommendation and have revised the conclusion to explicitly link our findings to SDG 6 (Clean Water and Sanitation).*

7. Authors indicated that “The survey was supplemented with field notebooks and cameras to aid direct field observations.” I did not see results reflective of that, or at least images/ pictures of observations from the field.

***Response:*** *We apologize for the oversight. We have now incorporated images from our field observations into the method section.*

8. In the discussion from line 6756 provide more specific details of the studies you are engaging with……eg. For example, a study in Ghana found that…. where exactly in Ghana?…

***Response:*** *We have revised the discussion to include more specific details regarding the studies referenced. For example, we now explicitly mention the regions or cities in Ghana where particular studies were conducted to allow for greater clarity and contextual relevance.*

**9. Overall Recommendation:** The paper addresses a critical issue in urban sanitation and provides valuable insights into the determinants of toilet ownership among property owners in Ghana. With some revisions of suggested areas for improvement, the manuscript could make a significant contribution to the field and be a valuable resource for policymakers. I recommend **minor revisions** before the manuscript is considered for publication.

***Response:*** *We sincerely appreciate the positive evaluation of our manuscript. We have addressed all the points raised to the best of our ability and believe that the revisions strengthen the overall quality and clarity of the paper.*
